# Exploration of the varieties differences on the volatile and non-volatile metabolites of *Alpinia galanga* and *Myristica fragrans* utilizing electronic sensing evaluation and untargeted metabolomics analysis

**DOI:** 10.1016/j.fochx.2025.102514

**Published:** 2025-05-09

**Authors:** Haijiao Lin, Xiangmin Li, Zengyi Song, Yu Liu, Zijia Li, Qingyun He, Binbin Wei, Ziwen Wang

**Affiliations:** a School of Pharmacy, China Medical University, No.77 Puhe Road, Shenyang 110122, PR China; b Department of Radiology, Shengjing Hospital of China Medical University, No.36 Sanhao Street, Heping District, Shenyang 110001, PR China; c Department of Oncology, Shengjing Hospital of China Medical University, No.36 Sanhao Street, Heping District, Shenyang 110004, PR China

**Keywords:** Untargeted metabolomics, Antioxidant activity, HS-GC–MS/MS, *E*-nose, Cardamom

## Abstract

There are differences in flavor, efficacy, and price between the cardamom varieties *Alpinia galanga* (H) and *Myristica fragrans* (R). It is crucial to distinguish their bioactivities and metabolites, given their high economic value and dietary frequency. This study determined total phenolic content (TPC), total flavonoid content (TFC), and in vitro/cellular antioxidant activities of R and H. Their volatile/non-volatile metabolites were analyzed via electronic sensing (*E*-nose, HS-GC–MS/MS) and untargeted metabolomics (UPLC-ESI-QTOF/MS^E^). Results demonstrated that R exhibited superior antioxidant capacity to H, with both correlating positively with TPC and TFC. A total of 195 non-volatile metabolites were identified and Malabaricone C, alkyl-DHAP, and (*R*)-Shinanolone were the key components for distinguishing them. *E*-nose showed W1C, W2W and W5S sensors were more sensitive. Furthermore, 219 kinds of volatile metabolites were identified, among which 14 key differential volatile components were screened out. The study established a theoretical basis for differentiating and characterizing both cardamoms.

## Highlights

• *Alpinia galanga* and *Myristica fragrans* were compared and comprehensively for the first time.
• This study presented the first multi-omics characterization of two cardamom species.
• *Myristica fragrans* showed superior antioxidant activity compared to *Alpinia galanga.*
• The e-nose successfully discriminated between *Alpinia galanga* and *Myristica fragrans*.

## Graphical abstract

**Figure:**
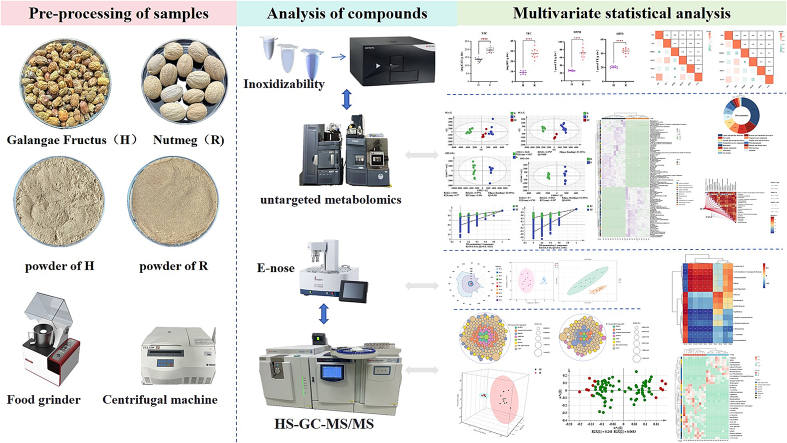

## 1 Introduction

As integral components of global gastronomy, spices function as gustatory enhancers, aromatic emitters, and phytotherapeutic agents, while historically playing an important role in civilizations, explorations, and commerce. Cardamom, a perennial aromatic herbaceous species, is known as the king of all spices. Its unique aroma components are regarded as the third most luxurious spice, surpassed only by *Crocus sativus* and *Vanilla planifolia*, and it is hailed as the “queen of spices” in commercial valuation (Wanna et al., 2024). *Myristica fragrans* (R) and *Alpinia galanga* (H) have been widely used in daily life. *Myristica fragrans* is commonly known as “nutmeg” and *Alpinia galanga* as “galangal”. Nutmeg was characterized by a dense and firm texture, smooth surface, high oil content, and a pungent aroma upon fracturing, while galangal exhibited a reddish-brown surface, plump morphology, and a strong aromatic odor (X. Chen, Feng, et al., 2022). Nutmeg contained volatile oils and lignans, such as α-pinene and β-caryophyllene, which exerted antioxidant effects by inhibiting lipoxygenase (LOX) activity and reducing the production of oxidative end-products like malondialdehyde (MDA). Extracts of nutmeg demonstrated anti-inflammatory, antimicrobial, antitumor, digestive-enhancing, and neuroprotective properties (Sreedharan et al., 2023). In contrast, galangal extracts mitigated oxidative damage through modulation of prostaglandins (6-keto-PGF1α) and vascular endothelial growth factor (VEGF) (Qin et al., 2018). Additionally, galanga exhibited anti-inflammatory, antimicrobial, and digestive-promoting activities. However, there were little research on the components and related active effects of galanga and systematic comparative metabolomic analyses of the two species and their functional divergence remained unreported. Their post-pulverization morphological convergence frequently leads to challenges in organoleptic differentiation and accidental substitution, necessitating comparative phytochemical profiling of their bioactive constituents for quality assurance protocols.

Untargeted metabolomics has been widely used in food research (Yan & Xu, 2018). Untargeted metabolomics serves dual analytical functions by delineating multifarious metabolite signatures while enhancing analytical breadth in compound detection. Ultra-high performance liquid chromatography-quadrupole time-of-flight mass spectrometry (UPLC-ESI-QTOF/MS^E^) constitutes an advanced analytical platform, providing foundational infrastructure for these highly efficient detection methods (Zhang et al., 2024). Organoleptic quality constitutes a determinant factor in food science that directly affects the aroma and thus influences consumers' preferences. Instrumental analysis techniques have become an indispensable part of food flavor analysis. Electronic nose (*E*-nose) analysis is a more cost-effective option, exhibiting rapid analytical processing, non-destructive analytical characteristics, easy to fabricate (Singh & Gaur, 2023). The application of the E-nose can simulate human olfaction through its excellent sensitivity, rapidity, simplicity, non-destructiveness and pollution-free characteristics, thereby ensuring quantifiable objectivity in food organoleptic profile characterization. As another technology for detecting flavor characteristics, headspace gas chromatography–mass spectrometry (HS-GC–MS/MS) is simple and practical. The system automates volatile organic compound (VOC) extraction from headspace vials followed by direct gas chromatography system injection, eliminating extraction head replacement requirements between analyses; this operational continuity facilitates uninterrupted batch processing while enhancing analytical throughput in food flavor profiling (Wei et al., 2023).

This investigation constitutes the inaugural comparative characterization of galangal and nutmeg, employing comprehensive phytochemical profiling that included total phenolic content (TPC), total flavonoid content (TFC), in vitro antioxidant capacity, cellular antioxidant activity (CAA), and chromatic parameter analysis (L^⁎^, a^⁎^ and b^⁎^). Furtherly the composition of non-volatile metabolites were compared through untargeted metabolomics analysis on the basis of UPLC-ESI-QTOF/MS^E^, aiming to explain the health benefits of edible legumes from the perspective of plant metabolism. The integration of multiple analytical techniques represents a prospective trend in food flavor analysis and can provide more detailed, comprehensive and reliable information. In addition, this study also integrated electronic nose (*E*-nose) and HS-GC–MS/MS to conduct a comparative analysis of volatile substances in the two types of cardamom. In conclusion, these results will provide a basis of the theory for evaluating the nutritional value of the two types of cardamom, in order to advance the use of cardamom in food industry.

## 2 Materials and methods

### 2.1 Reagents and instrument

Sodium formate (CAS No: 141–53-7, ≥99 %), leucine enkephalin (CAS No: 58822–25-6, ≥97 %), methanol (MeOH), ethanol (EtOH), dimethyl sulfoxide (DMSO), gallic acid, trolox, rutin, and Folin-Ciocalteu reagent. All other chemicals were of analytical grade or higher, sourced from Shanghai Yuanye Biotechnology Co, Ltd. (Shanghai, China).

### 2.2 Extraction of plant materials and samples

Cardamom (*n* = 10) was all purchased through online channels. See (Table. S3) for cardamom detail. Firstly, the purchased samples were milled into powder using a food grinder, sieved a 60-mesh sieve. Next, 1 g of powder was taken and dissolved in 5 ml of 70 % methanol. It was sonicated in a 500 W ultrasonic bath at room temperature for 10 min. After centrifugation (12,000 *g*, 4 °C, 10 min), the supernatant was retained. Repeat the operation three times and mix the clarified liquid. Store the extracted samples in a refrigerator at 4 °C overnight to precipitate the proteins. The next day, after the samples were taken out from the refrigerator and centrifuged. 20 samples extract were stored at −20 °C.

### 2.3 Determination of basic components

#### 2.3.1 TPC

The determination of TPC was slightly modified based on the method used in (Altuntas & Korukluoglu, 2024). The results were presented on milligram equivalents of gallic acid per gram of dry weight (mg GAE/g dw). See Supplementary Data 1 for experimental details.

#### 2.3.2 TFC

The determination of TFC was slightly modified according to the method used in (Liu et al., 2024). The results were presented on milligram equivalents of rutin per gram of dry weight of the sample (mg RE/g dw). See Supplementary Data 1 for experimental details.

### 2.4 In vitro antioxidant activity detection

DPPH (2,2-diphenyl-1-picrylhydrazyl) and ABTS (2,2′-Azino-bis (3-ethylbenzthiazoline-6-sulfonic acid)) radical cation scavenging and Ferric reducing antioxidant power (FRAP) were measured according to (Wang et al., 2024). By standardizing standard curve of the Trolox, the results were presented on Trolox equivalents (TE) per 1 g of sample (μmol TE/g dw). See Supplementary Data 1 for experimental details.

ACI is a commonly used computational metric (Lin et al., 2022). Score = (sample score / best score) × 100 %. The ACI is computed by the average index score of five results (TPC, TFC, DPPH, ABTS and FRAP) to comprehensively measure the antioxidant capacity.

### 2.5 Cellular antioxidant activity (CAA) assay

#### 2.5.1 Cell culture

Caco-2 cells were growed in MEM medium and put in a 5 % CO_2_ cell incubator at 37 °C. When the cells reached about 80 %, the cells were applied to the experiment.

#### 2.5.2 CAA experiment

The CAA experiment was carried out with modifications based on the method described in (Shan et al., 2024). The experiment results were presented on μmol of quercetin equivalent (QE) per 100 g of dry weight (μmol QE/100 g dw), and the percentage of reduction (or CAAunit) was calculated. See Supplementary Data 1 for experimental details.

### 2.6 Color determination

The color analysis was conducted with reference to previous methodologies (Chen et al., 2024) incorporating modifications. Chromatic parameters of cardamom samples were quantified using a YS6010 spectrophotometer (Sanen Time, Guangdong, China) through triplicate surface measurements per specimen. The CIELAB color space parameters (L^⁎^ for lightness, a^⁎^ for red-green axis, and b^⁎^ for yellow-blue axis) were determined to characterize the color profile. Standardized illumination conditions (D65/10° illuminant/observer) were implemented to simulate diffuse daylight observation on a white substrate, thereby optimizing measurement fidelity and reproducibility throughout the experimental process.

### 2.7 Untargeted metabolomics data acquisition based on UPLC-ESI-QTOF/MS^E^

The samples were tested taking advantage of the combination of a Xevo G2-XS QTOF-MS (Waters, Milford, USA) and a Water ACQUITY UPLC system. At 30 °C, 2 μL of the sample solution was injected into an ACQUITY UPLCTM BEH column (Waters, Milford, USA) at a flow rate of 0.4 mL/min. The mobile phase consisted of 0.1 % formic acid aqueous solution (solvent A) and 0.1 % formic acid acetonitrile solution (solvent B), with an elution time of 16 min and a flow rate of 0.4 mL/min. The gradient elution was designed as follows: 0 min, 5 % B; 2 min, 10 % B; 10 min, 70 % B; 12 min, 95 % B; 14 min, 95 % B; 14.1, 95 % B; 16 min, 5 % B. The QTOF worked in ESI^+^ and ESI^−^ modes. Quality control (QC) samples were prepared by mixing equal amounts of all the test samples and were repeated after every five test samples to assess the stability of the system.

### 2.8 Identification of compounds

A customized internal compound library with A total of 856 compounds were created through Progenesis SDF Studio. The raw data files of the UPLC-ESI-QTOF/MS^E^ analysis were collected using Masslynx software (Waters, Manchester, UK) and imported into Progenesis QI 2.3 software (Waters, Manchester, UK). Untargeted identification through the internal library and the HMDB database. According to the parameters of decreasing significance, the tolerance limits mass errors of precursor ions and fragments (All of them are 10 ppm); similarity degree of isotopes (>80 %); score (>30); fragment score (produced by the software) were considered. All mass spectrums were examined to guarantee that the fragments came from a single compound. More than 80 % of the compounds identified in each sample were regarded as initial appraisal.

### 2.9 *E*-nose analysis

Odor analysis of cardamom was carried out using a metal oxide semiconductor gas sensor-based electronic nose (*E*-nose, PEN 3 Airsense Analytics GmbH, Schwerin, Germany). It consists of 10 semiconductor sensors, each with specific specificity (Table. S2), which helps to accurately identify different categories of volatile compounds.

0.5 g of cardamom powder was taken and put in a 100 ml beaker. The beaker was sealed with double-layer plastic wrap and left to stand for 3 h before being tested on the machine. The injection needle was directly pluged in the sealed beaker including the sample, and the E-nose was utilized to launch the test. The time of sampling was 1 s per group; the time of self-cleaning the sensors was 80 s; the time of sensor zeroing was 5 s; the time of sample preparation was 5 s; the rate of injection flow was 400 ml/min; and the time of analysis sampling was 80 s.

### 2.10 Volatile compounds analysis based on HS-GC–MS/MS

The volatile ingredients were tested by HS-GC–MS/MS, using the Agilent 7890B gas chromatograph coupled with the Thermo Orbitrap Exploris GC mass spectrometer. Accurately weigh 1 g of cardamom powder and transfer into 10 mL headspace. The incubation temperature of the headspace sampler was 100 °C, the equilibration time was 10 min, and the desorption time was 4 min. The analysis was carried out using a gas chromatograph-mass spectrometer furnished with a 30 m × 0.25 mm × 0.25 μm TG-5MS quartz capillary column. Set helium (99.999 %) with a constant flow rate of 1 mL/min as the carrier gas to separate the volatile compounds. Headspace vial pressure: 90 kPa; pressure of the quantitative loop: 70 kPa; Temperature of the quantitative loop: 95 °C; Injection duration: 0.5 min; Inlet temperature: 200 °C. Temperature programming of the chromatographic column: The initial temperature was 40 °C, held for 2 min; heated to 90 °C at a heating rate of 8 °C·min^−1^ and held for 4 min; heated to 180 °C at a heating rate of 6 °C·min^−1^ and held for 5 min; heated to 260 °C at a heating rate of 15 °C·min^−1^ and held for 5 min; then run for 3 min with a split ratio of 10:1. Transfer line temperature: 230 °C; Column temperature: 230 °C. Mass spectrometry conditions: electron impulse ionization source (EI), ion source temperature 230 °C, scanning time from 0.5 to 43 min, scanning range from 30 to 600, resolution 30,000. The mass spectrometry data were contrasted with the National Institute of Standards and Technology (NIST) 17.0 L standard mass spectrometry search database (matching >80 %) to determine the volatile ingredients in the samples. The volatile components were qualitatively analyzed based on ratio of peak areas: a single volatile compound to the total peak area of all volatile compounds (% relative content) (Li et al., 2025).

### 2.11 Statistical analysis

Principal component analysis (PCA) and orthogonal partial least squares discriminant analysis (OPLS-DA) models were performed by Simca-p 14.1 software (SIMCA Imola s.c., Imola, Bologna, Italy), and the total ion chromatograms were plotted by Origin software (OriginLab Corporation, Northampton, Massachusetts, USA). Statistical analyses, including one-way ANOVA and homogeneity of variance tests, were carried out utilizing SPSS 18.0 (SPSS-IBM Chicago, IL, USA). Post hoc comparisons were made using the Duncan's multi-range test, with the remarkableness level set at *P* < 0.05. The data were presented on mean ± standard deviation.

## 3 Results and discussion

### 3.1 Total phenol and total flavone content

**Fig. 1:**
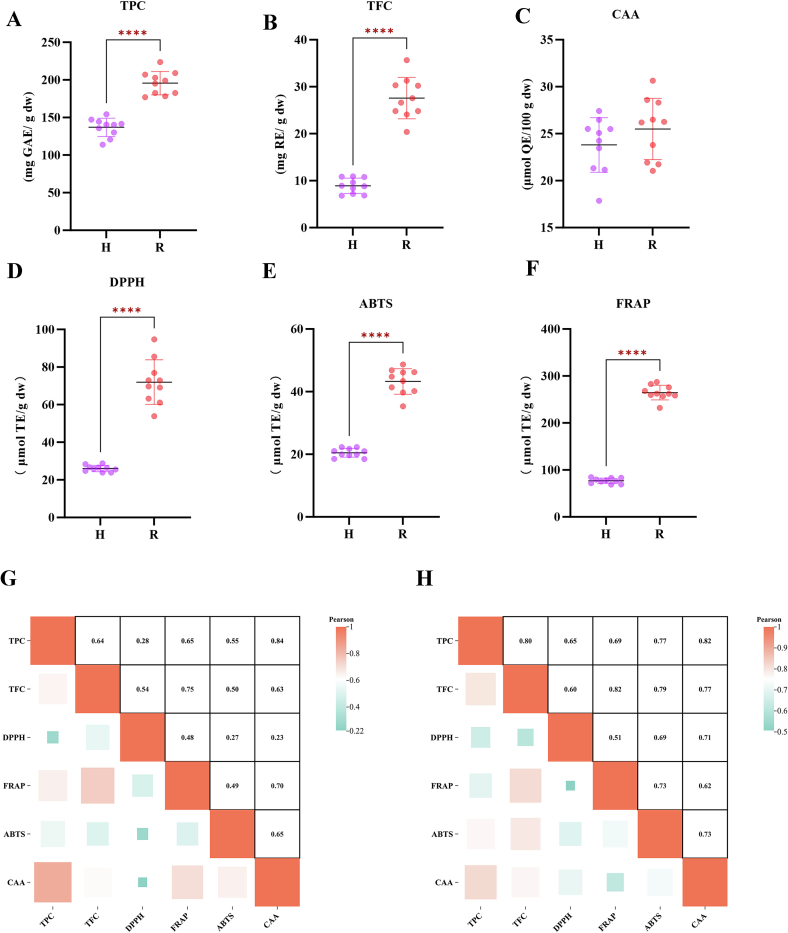
TPC (A), TFC (B), antioxidant activity CAA, DPPH, FRAP, ABTS (C—F); pearson correlation analysis of antioxidant activity in group *Alpinia galanga* (G) and *Myristica fragrans* (H). **** indicates a very significant correlation (*P* < 0.0001).

As shown in Fig. 1A, for TPC and TFC, the TPC of nutmeg was higher than that of galangal in all samples. The specific contents of each group were detailed in Table.S1. The TPC content of galangal ranged from 113.92 ± 1.82 to 147.10 ± 0.88 mg GAE/g dw, while TPC of R ranged from 176.89 ± 0.94 to 223.76 ± 5.56 mg GAE/g dw. In the comparison of TPC as shown in Fig. 1B, the TFC of R was higher than that of galangal. The TFC content of galangal ranged from 6.82 ± 0.59 to 10.92 ± 0.57 mg GAE/g dw, and the TFC content of R ranged from 20.38 ± 0.72 to 35.66 ± 1.22 mg GAE/g dw. Compared with galangal, nutmeg is a better source of flavonoid compounds, and the TFC content of R were more than two times higher than that of galangal.

### 3.2 Analysis of antioxidant capacity

Using different analytical methods to estimate the total antioxidant activity is of great importance for understanding the overall antioxidant potential of food matrices. Fig. 1 C, D and E evaluated the antioxidant activities of two types of cardamom against DPPH, ABTS and FRAP. The specific contents of each group were detailed in Table. S1. In the DPPH and ABTS experiments, the all dates displayed that the free radical clearing ability of galangal was lower than that of nutmeg. In DPPH, galangal was 23.87 ± 1.41 to 28.86 ± 2.42 mg GAE/g dw, while nutmeg was 61.13 ± 5.60 to 61.13 ± 5.60 mg GAE/g dw; in ABTS, galangal was 18.46 ± 1.68 to 22.28 ± 0.54 mg GAE/g dw, and nutmeg was 35.31 ± 0.63 to 48.66 ± 2.74 mg GAE/g dw. The chelating ability of cardamom to Fe^2+^ was evaluated by FRAP, and the results showed that galangal was 68.91 ± 2.34 to 84.63 ± 3.22 mg GAE/g dw, which was far lower than that of nutmeg (232.30 ± 3.55 to 287.09 ± 8.68 mg GAE/g dw). Overall, nutmeg was a strong antioxidant. There were not only significant differences in appearance between galangal and nutmeg, but also remarkable differences in antioxidant properties, which may imply the underlying differences in their metabolite ingredients and functions of biology.

The Pearson relevantion coefficients of TPC, TFC, DPPH, ABTS and FRAP were shown in Fig. 1 H and G. The TPC and TFC of galangal and nutmeg are highly positively correlated (0.64 and 0.80), indicating that the activities of the two may represent similar compounds. The correlation analysis between TPC and TFC with DPPH, FRAP and ABTS displayed that the TPC and TFC of nutmeg were extremely significantly positively correlated with DPPH, ABTS and FRAP respectively, and the Pearson correlation coefficients are all >0.5, indicating that the antioxidant capacity of nutmeg mainly comes from phenolic compounds and flavonoid compounds; the TPC and TFC of H were extremely significantly positively correlated with FRAP and ABTS respectively, and the Pearson correlation coefficients were all >0.5, and were positively correlated with DPPH. It indicated that the antioxidant capacities of these two types of cardamom may mainly come from phenolic and flavonoid compounds. In addition, almost all reports showed a high correlation between FRAP and ABTS, which was consistent with our results. The correlation between ABTS and FRAP was 0.49 in galangal and 0.73 in nutmeg. The specific data of ACI were shown in Table.S1. The average value of ACI of H and nutmeg differs by more than three times, indicating that there was a remarkable difference in the antioxidant capability in vitro between these two types of cardamom.

### 3.3 Cellular antioxidant capacity

CAA can represent the ability to scavenge peroxyl radicals and the accessibility of cell absorption, dispersion, and metabolism. According to the CAA resulted in Fig. 1 F, the contents of each group were shown in Table.S1, and both galangal and nutmeg showed cellular antioxidant activity. The value of CAA in H was 17.87 ± 1.48–27.41 ± 0.76 μmol QE/100 g dw, which was lower than that in nutmeg (21.05 ± 1.67–30.64 ± 0.48 μmol QE/100 g dw). In the correlation analysis between CAA and the in vitro antioxidant activity experiments of DPPH, ABTS, and FRAP, either galangal (Fig. 1 H) or nutmeg (Fig. 1 G) it was positively relevant with all of them, and *P* > 0.5. In the correlation between CAA and TPC and TFC, it was extremely significantly positively correlated (*P* > 0.6). These findings demonstrated consistency with the in vitro antioxidant activity measurements. Consequently, untargeted metabolomic profiling will be conducted to characterize compounds in both cardamom species.

### 3.4 Color analysis

Color analysis remains one of the most crucial subjects in the food industry. Psychophysical studies have confirmed that color perception influences taste perception and consumption patterns (Long et al., 2024). Through CIELAB measurements, the 20 cardamom samples were clearly classified into two groups based on their color values. In this system, L* represents lightness (0–100 scale), a* indicates the red-green component, and b* denotes the yellow-blue component. As shown in Fig. S4 A and B, group nutmeg exhibited L* values ranging from 44.26 to 47.29, while those in group galangal were significantly higher (62.69–67.36), indicating lighter coloration in the latter. The a* values, reflecting red intensity, measured 53.27–56.95 for group nutmeg versus 70.77–77.15 for group galangal, demonstrating stronger reddish characteristics in group galangal. Similarly, group nutmeg showed elevated b* values (82.75–89.59) compared to group galangal (72.46–76.83), suggesting higher yellow components. Three-dimensional scatter plots further confirmed distinct separation between the two groups (Fig. S4 C). Collectively, group nutmeg exhibited significantly higher chromatic intensity and greater visual saliency compared to other experimental groups. This analysis precisely determined the color characteristics of both cardamom types, facilitating deeper understanding of their sensory properties.

### 3.5 Analysis of untargeted metabolomics

#### 3.5.1 Identification of metabolites

**Fig. 2:**
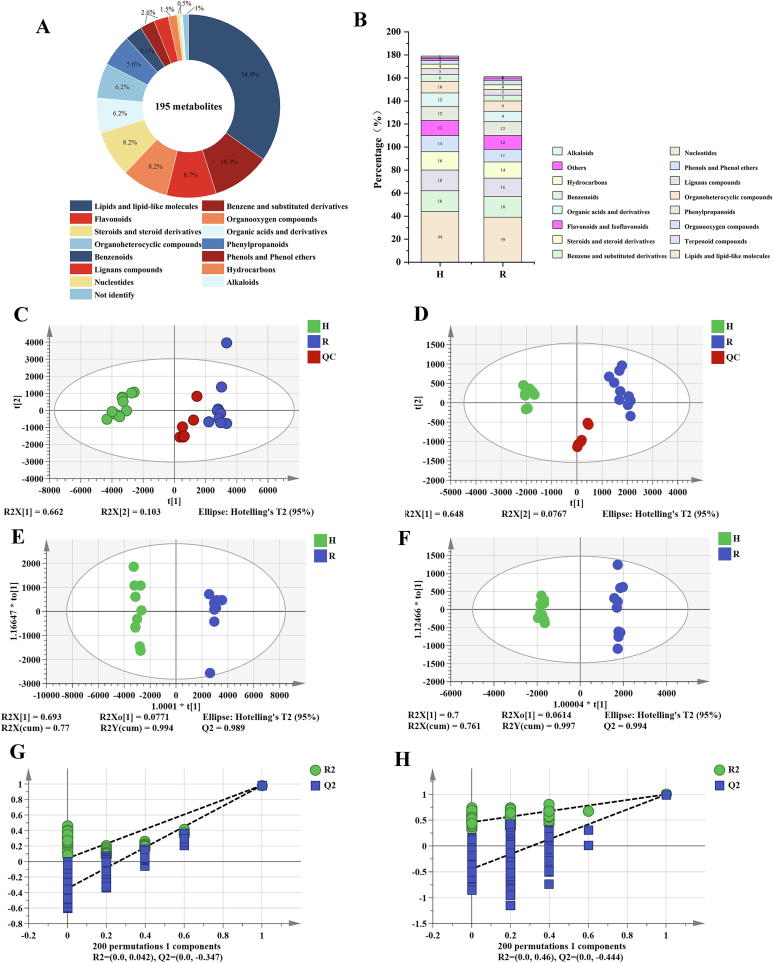
Classification of compounds identified by *Myristica fragrans* and *Alpinia galanga* (A); a total of 195 metabolites identified by metabolomics (B); PCA diagram in untargeted metabolomic positive (C) and negative (D) ion mode; OPLS-DA map in untargeted metabolomic positive ion mode (E) and negative (F) ion mode; permutations diagram in positive ion mode (G) and negative ion mode (H).

The results of the UPLC-ESI-QTOF/MS^E^ dates showed that the total ion chromatograms (TIC) of the QC group correctly overlapped in both ESI^+^ and ESI^−^ modes (Fig. S1). In the PCA diagram, the metabolites in the QC samples were clustered and separated from the sample plots, and the QC was located at the center of the PCA axes, which revealed the precision and stability of the metabolomics data in the study. Peak extraction and alignment in the chromatographic analysis yielded 10,288 positive peaks and 10,045 negative peaks, and the signal responses of these aligned peaks were used to evaluate the reproducibility of the instrument. By compared with domestic and foreign databases, simultaneously, the Human Metabolome Database (HMDB) (http://www.hmdb.ca/) database was used to search for and identify metabolites. A total of 195 metabolites were identified, and these metabolites were further classified into 16 categories, mainly including 48 lipid and lipid-like molecules, 20 benzene and substituted derivatives, 20 terpenoids, 17 flavonoids, 16 organic oxygen compounds, 16 steroids and steroid derivatives, 12 organic acids and their derivatives, 12 organic heterocyclic compounds, 11 phenylpropanoids, 6 stilbenoids, 5 phenols and phenol ethers, 5 lignans, 3 hydrocarbons, 1 nucleotide, 1 alkaloid, and 2 unidentified ones (Fig. 2 B). Among them, 179 were identified in H and 161 were identified in R (Fig. 2 A). See Supplementary Data 2 for specific non-volatile metabolites.

#### 3.5.2 Related active effects of compounds

In this study, the most abundant compounds identified were lipids and lipid-like molecules. Monoacylglycerides were typically added in small quantities to commercial foods as emulsifiers. Suberic acid, derived from the metabolic breakdown of oleic acid, was identified in both cardamom species. Heptanoic acid, detected exclusively in the nutmeg group, served as a precursor for synthesizing ethyl heptanoate, a compound widely used in flavoring agents and artificial fragrances. Triglycerides of heptanoic acid were utilized as nutritional supplements under specific medical conditions. 4-Hydroxynonenal (HNE), one of the primary end products of lipid peroxidation detected in both cardamom species, has been shown to participate in signal transduction and can influence cell cycle events in a concentration-dependent manner (Guo et al., 2024). Oleic acid, which was identified in both cardamom species, was the most widely distributed fatty acid in nature and the most abundant in human adipose tissue. It was utilized in the production of surfactants, soaps, plasticizers, and emulsifiers for food and pharmaceutical applications. Most flavor compounds were primarily obtained through the auto-oxidation or enzymatic oxidation of unsaturated fatty acids, particularly oleic acid (Al-Dalali et al., 2024).

The second group of ubiquitous metabolites was composed of terpenoids. L-Menthyl acetoacetate was recognized as one of the most important flavoring chemicals with extensive applications. (+)-Limonene, a naturally occurring monoterpene detected in both cardamom species, demonstrated lower aquatic toxicity than conventional solvents (n-hexane) and found broad applications in food flavoring, cosmetic fragrances, and aromatherapy. (+)-Limonene and its derivatives demonstrated potential antitumor activity by activating caspase-family proteases through the mitochondrial pathway, thereby promoting tumor cell apoptosis. Additionally, they exhibited neuroprotective effects by upregulating antioxidant enzymes including heme oxygenase-1 (HO-1), which mitigated neuronal oxidative stress damage (Pagliaro et al., 2023). As a multifunctional biosolvent, (+)-limonene exhibited significant potential in green chemistry and circular economy, with its core advantages lying in efficiency, safety, and sustainability (Pagliaro et al., 2023). Perillic acid (the oxidative metabolite of limonene), detected exclusively in group nutmeg, exerted antitumor, anti-inflammatory, and antiviral effects through multiple pathways, including inhibition of protein prenylation, modulation of immune responses, and direct induction of apoptosis (Rolim et al., 2025). α-Terpineol possessed an agreeable clove-like odor and was a frequent component in perfumes, cosmetics, and flavoring agents. p-Cymene, the sole naturally occurring isomer detected in both cardamom species, served as a constituent of various essential oils, most frequently found in cumin and thyme oils. Oleanolic acid, a ubiquitous triterpenoid in the plant and herbal kingdom detected in both cardamom species, represented an integral component of human diets. This compound exhibited antioxidant properties, anti-inflammatory activity, immune-enhancing effects, hypoglycemic effects, and hepatoprotective functions (Yang et al., 2016).

The third group of ubiquitous metabolites was identified as flavonoids. Petunidin and pelargonidin, anthocyanins that typically constituting components of human diets, were not only regarded as food constituents but also considered therapeutic agents. These compounds inhibited the expression of inflammatory mediators (COX-2, TNF-α) and demonstrated physiological activities, including anti-inflammatory, antioxidant, antitumor, and neuroprotective effects (Ma, Han, et al., 2025). Epicatechin exhibited biological activities such as antioxidant, anti-inflammatory, and antiviral properties, while enhancing stability and bioavailability through covalent binding with proteins (Q. Y. Ma, Xu, et al., 2025). Apigenin, a plant-derived flavonoid, showed significant promise as a chemopreventive agent against skin cancer. Isorhamnetin, the methylated metabolite of quercetin, exhibited in vitro antioxidant activity and hepatoprotective effects. Besides, its antifibrotic function was mediated through inhibition of the TGF-β/Smad signaling pathway, which manifested as reduced phosphorylation levels of Smad2/3 and subsequent suppression of fibrotic biomarkers including α-smooth muscle actin (α-SMA), plasminogen activator inhibitor-1 (PAI-1), and collagen type I alpha 1 (COL1A1)(Scarlata et al., 2025). In this experiment, petunidin, pelargonidin, epicatechin, apigenin, and isorhamnetin were detected in both cardamom species.

In conclusion, galanga and nutmeg contained abundant bioactive compounds that provided health benefits and found extensive applications in critical domains including food industry, cosmetic formulations, and disease prevention strategies.

#### 3.5.3 Multivariate statistical analysis of metabolites

To evaluate the performance of untargeted metabolomics analysis, an unsupervised PCA model was applied. The principal components PC1 and PC2 accounted for 66.2 % and 10.3 % of the total variation in the positive ion mode (Fig. 2 C), and 64.8 % and 7.67 % of the total variation in the negative ion mode (Fig. 2 D), respectively, suggesting that these PCA models have good adaptability and acceptable predictability. To better visualize and understand the chemical differences between the galangal and nutmeg samples, a relevantion analysis was calculated on the metabolomic data (Fig. S3 A and B). The intra-group correlation was higher than the inter-group correlation, indicating that the large difference between the two cardamoms may be due to the difference in compound content.

OPLS-DA is a steady comprehensive analysis method that can precisely identify the key factors affecting the whole. In the OPLS-DA model, the samples were clearly divided into two groups, achieving robust explanatory and predictive capabilities. In the positive ion mode, R2X = 0.77, R2Y = 0.994, Q2 = 0.989 (Fig. 2 E); in the negative ion mode, R2X = 0.761, R2Y = 0.997, Q2 = 0.994 (Fig. 2 F). The identification capability indicators (R2X and R2Y) and the prediction capability index (Q2) were all >0.5, with the values of R2Y and Q2 both approach 1. For the OPLS-DA model, we performed 200 permutation tests in both positive modes and negative modes and conducted internal test of the model to prevent overfitting (Fig. 2 G and H). Both R2 and Q2 produced by stochastic permutation were higher than those of the raw OPLS-DA model, indicating that the OPLS-DA model was accurate and effective and can be used for the screening of compounds for identification.

### 3.6 Differential compound analysis

**Fig. 3:**
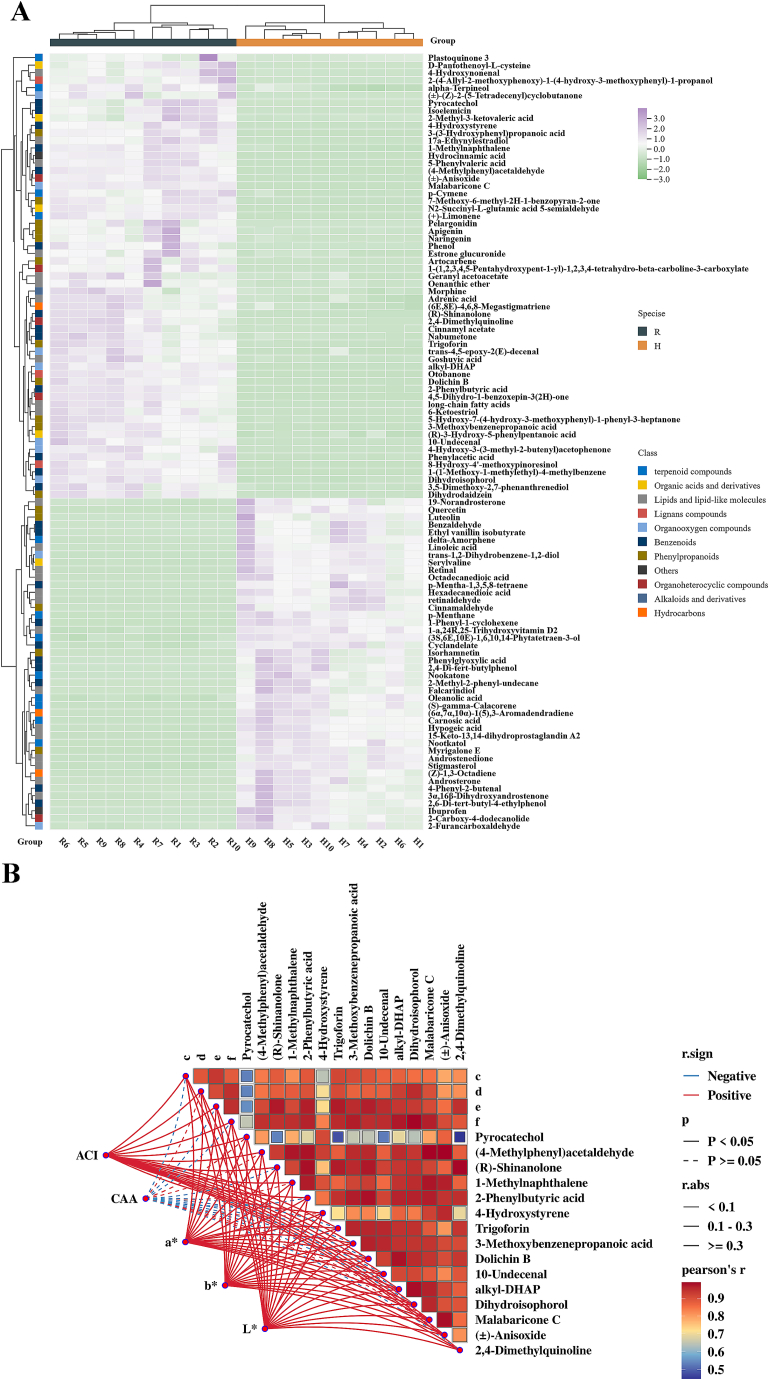
HCA of 103 different metabolites of two cardamoms(A); Correlation analysis of untargeted metabolome-differential compounds with CAA and ACI (B). e:5-Hydroxy-7-(4-hydroxy-3-methoxyphenyl)-1-phenyl-3-heptanone; f:1-(1-Methoxy-1-methylethyl)-4-methylbenzene; c:3,5-Dimethoxy-2,7-phenanthrenediol; d:4-Hydroxy-3-(3-methyl-2-butenyl) acetophenone.

Through multifactor analysis of the VIP values in the OPLS-DA model, based on the criteria of VIP > 1, fold change ≥2 or ≤ 0.5, and *p*-value <0.05, a total of 103 differential compounds were screened out from galangal and nutmeg. Excluding the two unidentified ones, these compounds can be classified into 10 types, namely 24 lipid and lipid-like molecules, 23 stilbenoids, 16 phenylpropanoids, 12 terpenoids, 9 organic oxygen compounds, 5 organic heterocyclic compounds, 5 organic acids and their derivatives, 3 hydrocarbons, 3 lignans, and 1 alkaloid and its derivatives. Among them, 93 metabolites (53 up-adjusted and 40 down-adjusted) were detected in the positive ion mode, 10 metabolites (6 up-adjusted and 4 down-adjusted) were detected in the negative ion mode. The distribution of different compounds was visualized by using hierarchical cluster analysis (HCA) and heat map (Fig. 3 A). Based on the clustering analysis of the situation, the two cardamoms could be divided into two groups, indicating significant discrepancies in their compositions. Additionally, due to the remarkable discrepancies in antioxidant abilities between the two types of cardamom, the relationships between stilbenoids, phenylpropanoids, and organic oxygen compounds among the differential metabolites and antioxidant activities were further surveyed.

#### 3.6.1 Benzoates

Phenols belong to a class of compounds called benzoates. Two phenolic substances (Phenol, Pyrocatechol) were screened out. Phenolic are non-enzymic antioxidant substances in plants that oppose free radical injury. They can straightforwardly clear H_2_O_2_ and decrease the accumulation of responsive oxygen species (ROS). It had been reported that the hydrogen-donating ability of phenolic acids in cardamom may lead to the scavenging or delaying of oxidation by transforming into OH- or O2·free radicals through single electron transfer (Kaur et al., 2024). Other authors had also found that phenolic substances have many health-promoting effects and possess high antioxidant activity (Alongi et al., 2025).

#### 3.6.2 Phenylpropane

A total of 16 phenylpropanoid and polyketide compounds were screened, including 6 flavonoid compounds (Apigenin, Isorhamnetin, Luteolin, Naringenin, Pelargonidin, Quercetin) and 2 isoflavonoid compounds (Dihydrodaidzein, Dolichin B). Isorhamnetin is a flavonoid. Besides exhibiting cardioprotective effected in H9c2 cardiomyoblasts under H, it also demonstrates antioxidant capacity against induced apoptosis and sensitizes hepatocellular carcinoma cells. Apigenin is a plant-derived flavonoid plentifully exist in fruits and vegetables and revealed the essence of PKCdelta in the process of Apigenin-induced apoptosis. Luteolin was a naturally occurring flavonoid (Shanmugam et al., 2020) and recognized for its potential in drug development, targeting various genes and pathways to exert anti-inflammatory effects. Dihydrodaidzein is an isoflavonoid compound that suppressed cell multiply and the formation of blood vessels in all kinds of cancer types and holds great promise in cancer of rectum. In conclusion, phenylpropanoid compounds possessed strong antioxidant activity and were beneficial to human health, which were consistent with the previous research findings (Zubova et al., 2024).

#### 3.6.3 Organic oxygen compounds

There were 9 organic oxygen compounds that had been discovered, including 7 carbonyl compounds (2-Furancarboxaldehyde, 4-Hydroxy-3-(3-methyl-2-butenyl) acetophenone, alkyl-DHAP, Dihydroisophorol, Malabaricone C, trans-1,2-Dihydrobenzene-1,2-diol, trans-4,5-epoxy-2(*E*)-decenal) and 2 alcohols and polyols ((±)-(Z)-2-(5-Tetradecenyl)cyclobutanone, 10-Undecenal). Malabaricone C was a diarylnonane bioactive compound that exists in the dried seed coat of cardamom and possesses strong scavenging and antibacterial activities. Malabaricone C inhibits SARS-CoV-2 entry and fusion by disrupting sphingomyelin distribution and lipid raft formation on the host cell membrane. With its broad-spectrum antiviral activity and high safety profile, it represents a promising candidate for COVID-19 therapeutics(Mutmainah et al., 2025).

### 3.7 Correlation analysis

Antioxidant capacity serves as a connection connecting various activities of biology. To explore the internal relationships, we conducted Pearson's r correlation analysis between the differential compounds and ACI、CAA as well as color value. So as to screen out the metabolites that contributed remarkably to biological activities, we selected those with a relevance coefficient *P* ≤ 0.05. It could be distinctly displayed in Fig. 3 B that 19 compounds (8 toluene-like compounds, 5 oxygen-containing organic compounds, 4 phenylpropanoids, 2 heterocyclic organic compounds) showed positive correlation with ACI and three color values (L, a, b*). 7 compounds (4-Hydroxy-3-(3-methyl-2-butenyl)acetophenone, Pyrocatechol, (4-Methylphenyl)acetaldehyde, 1-Methylnaphthalene, 4-Hydroxystyrene, Malabaricone C, (±)-Anisoxide) showed positive correlation with CAA, all with statistical significance. Intercorrelation analysis revealed that all 19 compounds had significant positive associations with each other, indicating their collective contribution to bioactive properties. As evidenced in Fig. S5 and Fig. S6, three metabolites (Pyrocatechol, 2,4-dimethylquinoline, and 10-undecenal) were not detected in Group galangal, while the remaining metabolites were identified in both cardamun species. In addition，group nutmeg exhibited significantly higher relative content of these compounds compared to group galangal, which explaineds its enhanced antioxidant capacity.

Pyrocatechol exhibited weaker positive correlations with other compounds, but demonstrated a significant positive correlation (*r* = 0.91, *P* < 0.001) with 4-Hydroxystyrene. Both compounds showed strong positive associations with ACI, CAA, and color parameters. This concentration-dependent synergistic relationship between Pyrocatechol and 4-Hydroxysteine mechanistically contributed to enhanced antioxidant efficacy and improved color attributes in galanga extracts. Both Pyrocatechol and 4-Hydroxystyrene contain phenolic hydroxyl groups capable of scavenging free radicals and reducing oxidative stress-induced cellular damage, thereby providing anti-inflammatory and antioxidant effects. Additionally, 4-Hydroxystyrene efficiently generates stable pyranoanthocyanins via condensation and aromatization pathways, offering critical theoretical support for industrial-scale natural pigment production (Guo et al., 2024). The highest concentrations of Malabaricone C, alkyl-DHAP, and (*R*)-Shinanolone were detected in Group nutmeg. These compounds exhibited highly significant positive intercorrelations (*r* > 0.91, *P* ≤ 0.05) and showed positive associations with in vitro antioxidant capacity and color indices, further confirming their synergistic dominance in enhancing antioxidant performance and chromatic characteristics. Malabaricone C, containing multiple phenolic hydroxyl groups, neutralizes free radicals via hydrogen atom donation to terminate oxidative chain reactions. Alkyl-DHAP serves as a core enzymatic catalyst for ether bond formation, demonstrating high efficiency and substrate specificity in ether lipid biosynthesis. The superior antioxidant capacity of group nutmeg compared to group galangal primarily stems from the cumulative effects of these three compounds.

The two screened organic oxygen compounds (2,4-Dimethylquinoline, (±)-Anisoxide) and 10-Undecenal were only detected in nutmeg. (±)-Anisoxide has been proven to exist in many spices (Huong et al., 2016)and was a flavor enhancer and a non-sensitizer. 2,4-Dimethylquinoline was utilized as an oxidizing agent, dehydrating agent, and catalyst in organic synthesis. Trigoforin and 10-Undecenal was often detected in herbs and spices, which was consistent with our research. 10-Undecenal was characterized by a distinctive coconut-like aroma and was recognized as an important natural compound in food flavorings. Both 10-undecenal and (±)-anisoxide found extensive applications in various industries, including cosmetics, perfumery, and the food sector. Color correlation analysis revealed that antioxidant-active compounds directly mediated chromatic alterations, consistent with established research findings (Bai et al., 2025). These findings revealed the intricate interactions among cardamom constituents, where total phenolic and flavonoid contents enhanced quality by directly affecting both coloration and antioxidant properties. These findings played a pivotal role in advancing the development of cardamom within the cosmetics, food, and pharmaceutical industries.

### 3.8 Analysis of *E*-nose

**Fig. 4:**
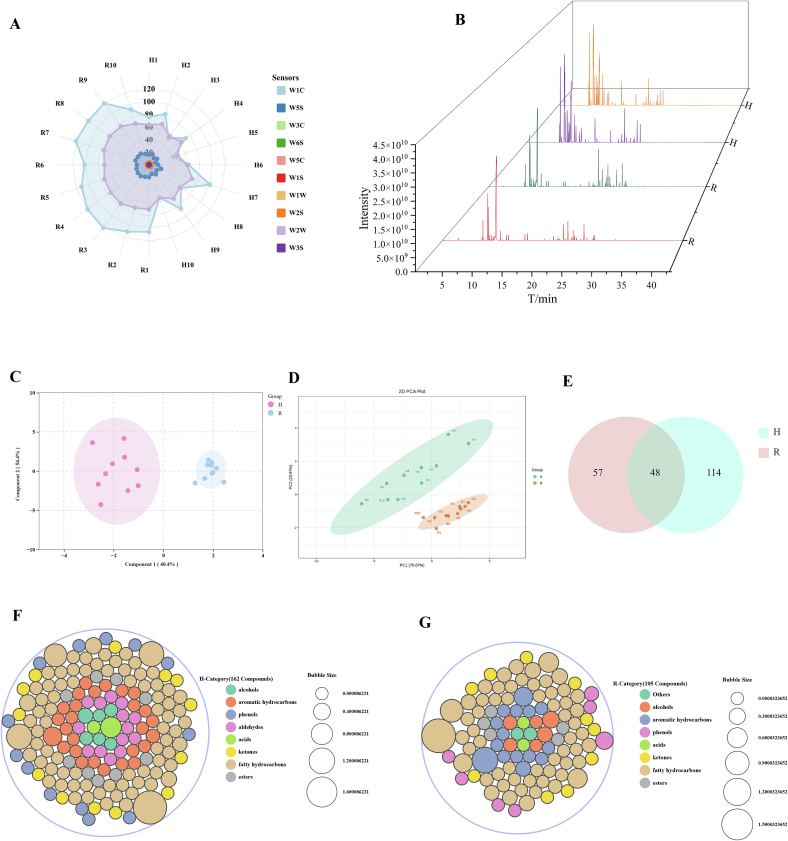
Radar map of the response of 10 electronic nose sensors (A); OPLS-DA score chart (C); fractions of PCA (D); total ion current chromatogram (TIC) of volatile compounds (B); venn diagram of volatile metabolites identified in H and R (E); bubble plot of volatile compounds identified in *Alpinia galanga* (F) and *Myristica fragrans* (G).

The e-nose is capable of detecting slight changes in samples, which may cause different responses from the sensors. To better visualize the analysis of the electronic nose, a radar chart was used to display the overall flavor differences between the two types of cardamom (Fig. 4 A). The values of sensors W1C, W2W, and W5S were relatively high in both galangal and nutmeg, indicating that the contents of benzene, nitrogen oxides, and aromatic components in galangal and nutmeg were relatively high, with high sensitivity. Moreover, it was obvious that the sensitivity of galangal to the sensors was relatively lower compared to that of nutmeg.

To classify the samples, PCA was used to determine the signal intensity of the electronic nose (Fig. 4 D), in order to emphasize the differences in volatile components. For the cardamom samples, the two principal components PC1 accounted for 70.67 % and PC2 accounted for 23.97 % respectively. The accumulated devotion rate of the first and second principal components reached 94.64 % (> 90.00 %), covering almost all the information of the samples and effectively characterizing the differences between the samples. In addition, the corresponding loading plot analysis showed the key sensors that caused these differences (Fig. S3 C), thus enabling the investigation of which type of gas played a major role in the process of sample differentiation. The sensors that contribute more to the first principal component were WIC, W2W, and W5S in sequence, and the sensor that contributes more to the second principal component was W2W. As shown in the figure (Fig. 4 C), the dispersion contribution rated of PC1 and PC2 were 40.4 % and 54.4 % separately, accounting for 94.8 % of the total variance in the OPLS-DA score plot.

The e-nose analysis successfully discriminated between cardamom varieties, though the derived volatile profiles exhibited limited comprehensiveness and analytical precision in compound characterization. It was essential to combine the electronic nose with HS-GC–MS/MS to contrast and analyze the differences between the two types of cardamom comprehensively, systematically and accurately at the small molecule level.

### 3.9 Identification of volatile compounds

The total ion current chromatogram (TIC) of volatile of galangal and nutmeg had been obtained by HS-GC–MS/MS (Fig. 4 B). A total of 219 volatile metabolites were detected in 20 sample groups, including 47.48 % aliphatic hydrocarbons, 15.98 % aromatic hydrocarbons, 9.13 % ketones, 8.22 % phenols, 6.39 % aldehydes, 6.39 % esters, 4.57 % alcohols, 0.91 % acids, and 0.91 % others. 162 compounds were appraised in galangal (Fig. 4 F) in total. 105 compounds were appraised in nutmeg (Fig. 4 G) in total. The Venn diagram showed that galangal and nutmeg had 48 substances in common (Fig. 4 E). See Supplementary Data 3 for specific volatile metabolites.

#### 3.9.1 Analysis of volatile compounds

The largest number of aliphatic hydrocarbons were appraised, taking up 47.48 % of the whole volatile substances. Hydrocarbons were the mainly formed through the homolytic cleavage of alkoxy groups in fatty acids. Interestingly, in the exploration of cardamom in other literature, the number of hydrocarbons not detected was not as many as in this experiment. This may be because during the steam distillation extraction process, hydrocarbons may be converted into corresponding esters, alcoholsand, aldehydes and other derivatives. Terpenes were part of of hydrocarbons and were widespreadly present in all kinds of plants, such as leschenaultii. Cardamun was found that the main terpenoid substances in cardamom include β-Pinene, α-Pinene, Sabinen, (−)-Terpinen-4-ol, Terpinen-4-ol, Methyleugenol. Unfortunately, in study, this component was not recorded in the nutmeg group, while these substances were all detected in the galangal group. It is speculated that the content of α-Pinene in our nutmeg sample group may be too low to achieve the detection level of the GC–MS apparatus. α-Guaiene, exclusively identified in Group galangal, was characterized as a sesquiterpenoid compound demonstrating multifunctional bioactivities: platelet aggregation inhibitory effects, plant metabolic regulation, and therapeutic potential against dental plaque formation, hyperglycemia, and allergic rhinitis symptomatology (Park et al., 2024). Aromatic hydrocarbons constituted 15.98 % of the volatile profile and imparted characteristic odor profiles to cardamom. Sabinene hydrate, which was detected in both types of cardamom, had the flavors of citrus, earth and wood, while isoeugenol, which was only detected in nutmeg, has a sweet and spicy odor (Spence, 2024). 2-Furanmethanol, which was only detected in galangal, is featured by coffee, moldy and sweet flavors (Choe et al., 2022). Phenols were essential aromatic compounds and weie the most abundant compounds in herbal spices. 18 kinds of phenolic volatile substances were identified.

Carbonyl compounds, including ketones and aldehydes, have the ability to promote and enhance flavors (Zhang et al., 2025). Relatively few alcohol compounds were detected, accounting for only 4.57 % of the total identified volatiles. Alcohols mainly came from the reduction of aldehydes, which were the products of lipid oxidation. Piperitenol detected in nutmeg were a non-protein product of lysine catabolism and had various functions of biolory such as antibacterial, partial anesthetic, anticancer, and neuromodulatory effects (Al-Rooqi et al., 2022).

The metabolites of esters are instable and can easily be converted into other substances. They were usually formed by the reaction involving alcohols and carboxylic acids produced by metabolism of lipid compounds. Esters produced by short-chain fatty acids have the taste of fruit, while those produced by long-chain fatty acids produce the taste of fat. Methyl cinnamate functions as a flavoring agent, a spice, an insect attractant, a volatile oil component, and an anti-inflammatory agent. Methyl linoleate was the fatty acid methyl ester of linoleic acid and was functionally related to linoleic acid. Relatively few volatile substances of acids were identified. One of the main metabolites of plants is organic acids, which were crucial for maintaining the value of nutrition and quality of foods. It was worth noting that in (Cruz et al., 2024), methoxy eugenol and elemicin were observed as the main components in nutmeg, but neither of them was found in our two cardamom samples. This could be attributed to factors such as chemotype, geographical location, plant collection season, plant development stage, climate, extraction technique, plant variety, and the plant part used, which could affect the biological activities of cardamom.

#### 3.9.2 Multivariate statistical analysis of volatile compounds

**Fig. 5:**
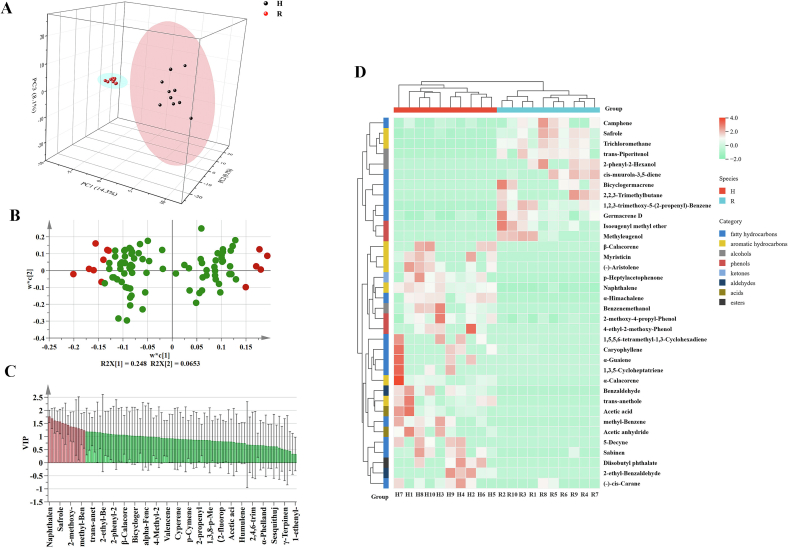
3D PCA diagram of volatile substances (A); Load diagram of volatile compounds (B); VIP diagram of volatile compounds (C); Cluster Analysis of 36 VIP > 1 (D).

**Fig. 6:**
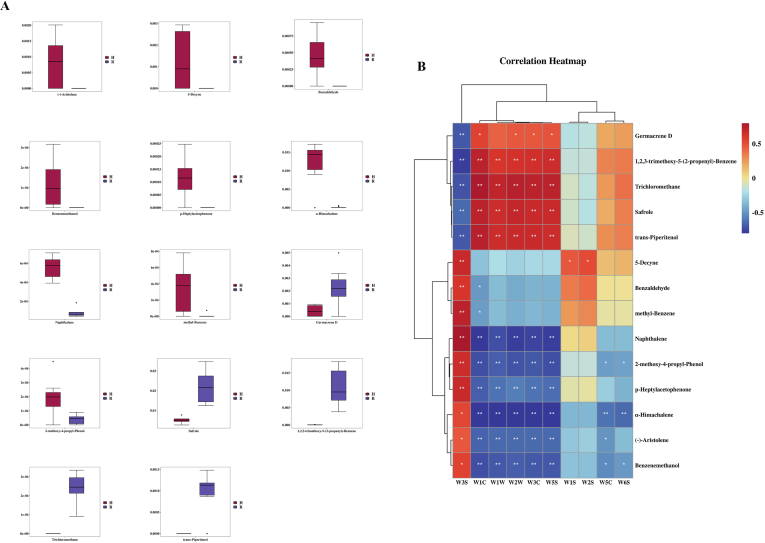
Boxplot of the relative contents of 14 key volatile substances in *Myristica fragrans* and *Alpinia galanga* (A)；Correlation analysis diagram of 14 characteristic volatile components of GC–MS and electric nose sensor(B). * indicates a significant correlation (P < 0.05), ** indicates a very significant correlation (P < 0.01), and *** indicates a very significant correlation (*P* < 0.001).

The 3D-PCA plot (Fig. 5 A) showed that PC1, PC2, and PC3 account for 14.3 %, 9.1 %, and 8.3 % of the total variance respectively. Each group of samples exhibited an obvious separation trend, indicating that there was remarkable distinction in the volatile metabolites between galangal and nutmeg. In the loading plot (Fig. 5 B), the farther a variable were from the center point, the greater its contribution to the classification. It could be clearly seen from the loading plot that a total of 13 volatile substances represented by red are farther from the midpoint. In Fig. 5 C, the red part were the volatile compounds with VIP > 1 and a total of 36 different compounds were screened. To clear visualization of the changes in volatile compounds with differences, the HCA was drawn (Fig. 5 D). Further screening of the differential volatile substances yielded 14 key differential volatiles with VIP > 1.2. The relative contents of these 14 key compounds in galangal and nutmeg were shown in (Fig. 6 A). (−)-Aristolene, 5-Decyne, Benzaldehyde, Benzenemethanol, and p-Heptylacetophenone were only identified in galangal, while trans-Piperitenol and Trichloromethane were only identified in nutmeg. The remaining 7 volatile substances were detected in both galangal and nutmeg. (−)-Aristolene were a sesquiterpene compound which could be used to distinguish the floral fragrance components of four Panax notoginseng flower species (S. Chen, Rui, et al., 2022), and it could also be used to distinguish galangal and nutmeg. (−)-Aristolene exhibited antifungal activity, while synthetic chemical fungicides were employed to safeguard stored grains against microbial deterioration. Benzaldehyde is an aldehyde which are usually formed through lipid oxidation. Due to their low flavor thresholds, they have a unique aroma at low concentrations. The compound was identified as a fragrance allergen and served as a chemical intermediate, demonstrating solubility in resin and lipid matrices. Benzenemethanol is an alcohol and is one of the four most commonly used antibacterial preservatives in biopharmaceutical injection formulations (Selaya et al., 2024). The compound acted as an anesthetic agent and topical pharmaceutical adjuvant. Upon contact with neuronal fibers, it induced sensory and motor paralysis in innervated regions. p-Heptylacetophenone is a long-chain ketone. Short-chain ketones have a taste of fat and burnt smell, while long-chain ketones emit a fragrance. *Trans*-Piperitenol is a p-menthane monoterpene compound. Trichloromethane historically served as a solvent, fumigant, and processing agent for resins, plasticizers, rubber chemicals, and fragrance dry-cleaning formulations. Additionally, it functioned as a chemical precursor in industrial synthesis. It had also been detected in chickpeas, red speckled kidney beans, and mung beans in previous studies (He et al., 2023). In summary, these 14 key volatile substances were the keys to distinguishing the two cardamom varieties.

The aforementioned analyses of non-volatile and volatile compounds in cardamom species demonstrated their multifunctional biological roles. Phytochemical constituents exhibited diverse pharmacological properties, including antimicrobial, anti-inflammatory, antioxidant, insecticidal, local anesthetic, anticancer, and neuromodulatory activities. In food science applications, these compounds served critical roles as flavor enhancers, aroma intensifiers, natural preservatives, and stabilizers for maintaining nutritional integrity and organoleptic properties in processed foods.

### 3.10 Correlation analysis between key odor compounds and sensors

To further analyze the differential volatile substances, a correlation analysi (Fig. 6 B) was conducted on the 14 key volatile metabolites and 10 sensors. All the sensors were divided into 4 classifications. The first group was W3S. The substances that had an enormously remarkable negative correlation (*P* < 0.01) with this type of sensor were trans-Piperitenol, Safrole, Trichloromethane, 1,2,3-trimethoxy-5-(2-propenyl)-Benzene, and Germacrene D. The other 9 key odor substances all had an enormously remarkable positive correlation (*P* < 0.01) with the W3S sensor. The second category included W1C, W1W, W2W, W3C, and W5S. The substances that had an enormously remarkable positive correlation (*P* < 0.01) with this type of sensor were trans-Piperitenol, Safrole, Trichloromethane, 1,2,3-trimethoxy-5-(2-propenyl)-Benzene. Only Germacrene D had a positive correlation (*P* < 0.05) with this type of sensor. The substances that had a positive correlation with this type of sensor were Benzenemethanol, (−)-Aristolene, α-Himachalene, p-Heptylacetophenone, 2-methoxy-4-propyl-Phenol, and Naphthalene, while methyl-Benzene and Benzaldehyde had a negative relevance (*P* < 0.05). The third category was W1S and W2S. Only 5-Decyne had a positive relevance (*P* < 0.05) with them. The fourth category was W5C and W6S, characterized by substances having only a remarkable negative relevance. This type of sensor had an enormously remarkable negative relevance (*P* < 0.01) with α-Himachalene, while Benzenemethanol and 2-methoxy-4-propyl-Phenol had a negative relevance (*P* < 0.05). In this type of sensor, (−)-Aristolene had a negative relevance (*P* < 0.05) only with W5C.

## 4 Conclusion

In conclusion, this study identified the basic nutritional components and metabolite compositions of two cardamom species (*Alpinia galanga* and *Myristica fragrans*). The antioxidant activity of *Alpinia galanga* in vitro and cellular was lower than that of *Myristica fragrans*, and both of them were positively correlated with TPC and TFC. This study conducted a comprehensive analysis of the antioxidant properties, non-volatile metabolites, intelligent sensory *E*-nose, and volatile components of the two types of cardamom for the first time. Overall, the different expressions of metabolites in different types of cardamom were reported, which may reveal the differences in their biological functions establishing quality assurance mechanisms and the potential applications of these cardamoms in the future.

## CRediT authorship contribution statement

**Haijiao Lin:** Writing – original draft, Methodology, Investigation. **Xiangmin Li:** Formal analysis, Data curation. **Zengyi Song:** Resources, Methodology. **Yu Liu:** Validation, Investigation. **Zijia Li:** Data curation. **Qingyun He:** Methodology. **Binbin Wei:** Writing – review & editing, Funding acquisition. **Ziwen Wang:** Writing – review & editing.

## Fundings

This study was supported by the 10.13039/501100001809National Natural Science Foundation of China (NO.81703519), 10.13039/501100005047Liaoning Province Natural Science Foundation (2023-MS-179), Shenyang City Science and Technology Project (No. 24-213-3-45).

## Declaration of competing interest

The authors declare that they have no known competing financial interests or personal relationships that could have appeared to influence the work reported in this paper.

## Data Availability

Data will be made available on request.

## Appendix A Supplementary data

Supplementary material 1 Image 1

Supplementary material 2 Image 2

Supplementary material 3 Image 3

Supplementary material 4 Image 4
