# Supplementary material for: Exploration of the varieties differences on the volatile and non-volatile metabolites of Alpinia galanga and Myristica fragrans utilizing electronic sensing evaluation and untargeted metabolomics analysis
Source: Food Chem X. 2025 May 9;28:102514. doi: 10.1016/j.fochx.2025.102514 (PMC12145681; doi:10.1016/j.fochx.2025.102514)
Supplement: Supplementary file 1 — Supplementary material 1 [file mmc1.docx]

**Exploration of the varieties differences on the volatile and non-volatile metabolites of *Alpinia galanga* and *Myristica fragrans* utilizing electronic sensing evaluation and untargeted metabolomics analysis**

**Determination of TPC, TFC and antioxidant activity**

The determination of TPC using Folin-Ciocalteu reagent, gallic acid was used as a standard. 200 μL of appropriately diluted samples were mixed with 1.5 mL of Folin-Ciocalteu reagent (diluted 1:5 in distilled water), vortexed and homogenized for 30 s, and incubated for 2 min at room temperature. Then, added 1.2 mL of sodium carbonate solution (7.5%, w/v) and incubated the samples for 60 min at room temperature in the dark. The absorbance of the samples and calibration curve standards were measured at 765 nm. A blank consisting of water and samples was used as a reference. Sample color blanks were checked using samples and water to exclude color interference from sample extracts. Data were calculated by comparing the standard curve (10-100 μg/mL gallic acid) with the absorbance of each sample. The results were expressed in milligram equivalent of gallic acid per gram of sample dry weight (mg GAE/ g dw).

TFC was determined by the spectrometric method, using rutin as a standard. In short, 2.7 mL of appropriately diluted extract was added to 150 μL of NaNO2 solution (5% w/v) and mixed. After 5 min, 150 μL of AlNO3 (10% w/v) was added. After 6 min, 2 mL of 1.0 M NaOH was added. The absorbance at 515 nm was measured by incubating for 10 min at room temperature in the dark. Data were calculated by comparing the standard curve (5-100 μg/mL rutin) with the absorbance of each sample. The results were expressed as milligram equivalents of rutin per gram of sample dry weight (mg RE/ g dw).

The antioxidant activity of the samples was assessed by DPPH radical scavenging activity assay with appropriate modifications. Briefly, 0.5 mL of appropriately diluted sample extract was mixed with 2.5 mL of freshly prepared DPPH radical methanol solution (80 μM). After standing in the dark for 2 h, the absorbance was measured at 515 nm. Trolox was used as a standard for creating calibration curves (20-200 μM). The results of antioxidant activity were expressed as μmol of Trolox equivalent antioxidant capacity per gram of the sample dry weight (μmol TE/g dw).

The ABTS assays were performed using a previous method with minor modifications. ABTS working solution was prepared by mixing potassium persulfate (2.45 mmol/L) and ABTS (7 mmol/L) (1:1, v/v) and incubated at room temperature and protected from light for 12-16 h. The working solution was then diluted with distilled water to obtain an absorbance value of 0.70 ± 0.02 at 734 nm. Next, 200 μL of the appropriately diluted sample solution was mixed with 2.8 mL of ABTS working solution. The mixture was then incubated in the dark at 25°C for 6 min and its absorbance at 734 nm was measured. Trolox was used as a standard for creating the calibration curve (30-300 μM). The results of antioxidant activity were expressed as μmol of Trolox equivalent antioxidant capacity per gram of the sample dry weight (μmol TE/g dw).

The ferric-reducing antioxidant capacity (FRAP) assay was based on a previous method with some modifications. The FRAP solution consisted of 2.5 mL 10 mM TPTZ solution (0.31 g TPTZ dissolved in 100 mL of 40 mM HCl), 2.5 mL FeCl3·6H2O water solution (20 mM) and 25 mL acetate buffer (0.3 M, pH= 3.6). The mixture was heated to 37°C before use. 200 μL of the appropriately diluted sample solution was mixed with 2.8 mL of FRAP working solution for 30 min at room temperature and its absorbance was measured at 593 nm. Trolox was used as a standard for creating the calibration curve (40-400 μM). The results of antioxidant activity were expressed as μmol of Trolox equivalent antioxidant capacity per gram of the sample dry weight (μmol TE/g dw).

**Determination of CAA**

Cells were plated (6 × 10⁵ cells/well) in 96-well black flat-bottom tissue culture treated plates and incubated at 37 °C for 24 - 48 h. After incubation, the growth medium was removed, and the cells were then washed with PBS to remove any non-adherent and dead cells.

The antioxidant (50 μL) and DCFH-DA (50 μM) solutions were added to each well in triplicate. After incubation at 37 °C for 20 min, the cells were washed twice with PBS. Then, 100 μL of AAPH solution (600 μM) was added to the wells, and the plate was immediately placed in a multi-functional microplate reader. The real-time fluorescence was initially read, and then the fluorescence was read every 5 min for 1 h, with an excitation wavelength of 485 nm and an emission wavelength of 538 nm. The control wells were treated with DCFH-DA and AAPH without antioxidants, while the blank wells were treated with DCFH-DA without AAPH and antioxidants. A standard curve of quercetin was established with the quercetin concentration (1 - 15 μM) on the horizontal axis and the CAAunit on the vertical axis. The final results were expressed as μmol of quercetin equivalent per 100 g of dry weight (μmol QE/100 g), and the percentage of reduction (or CAAunit) was calculated.

CAAunit = %reduction = (1 - AUCsample/AUCcontrol) × 100.

**Table. S1** Total phenolic content (TPC), total flavonoid content (TFC), in vitro antioxidant activity (DPPH, ABTS and FRAP), antioxidant potency index (ACI) and cellular antioxidant activity (CAA) of two species of cardamun and correlation coefficients each other.

| H | TPC | TFC | DPPH | ABTS | FRAP | ACI | CAA(μmol QE/  100 g dw) |
| --- | --- | --- | --- | --- | --- | --- | --- |
| H1 | 113.92±1.82d | 6.82±0.59d | 26.55±0.70a | 18.46±1.68ab | 68.91±2.34e | 32.00 | 17.87±1.48e |
| H2 | 147.10±0.88a | 10.92±0.57a | 26.59±3.53a | 22.28±0.54a | 82.95±1.07b | 39.83 | 27.41±0.76a |
| H3 | 140.49±2.02ab | 9.61±0.38b | 25.90±3.87a | 20.96±0.75b | 76.06±2.76cd | 37.33 | 25.50±0.75abc |
| H4 | 130.07±1.71c | 7.20±0.17d | 24.79±1.53a | 19.99±0.54ab | 71.98±1.07de | 34.13 | 23.46±0.56cd |
| H5 | 141.57±4.45ab | 8.82±0.75bc | 23.87±1.41a | 21.72±1.13ab | 75.45±2.26cd | 36.83 | 25.51±1.09abc |
| H6 | 120.74±1.18d | 6.86±0.21d | 26.37±0.26a | 18.52±0.99ab | 69.03±1.98e | 32.63 | 21.16±0.88d |
| H7 | 120.74±1.18d | 8.92±0.29bc | 25.57±0.80a | 19.74±0.48a | 77.88±0.95a | 34.74 | 21.332±0.76d |
| H8 | 140.38±11.49ab | 10.86±0.32a | 28.86±2.42a | 19.92±2.99b | 84.63±3.22ab | 38.82 | 25.08±2.95abc |
| H9 | 144.44±5.41ab | 8.40±0.19c | 28.40±2.19a | 19.65±1.69ab | 83.04±3.16ab | 37.48 | 26.47±1.46ab |
| H10 | 135.77±4.10c | 10.78±0.14a | 26.72±2.11a | 21.04±1.35ab | 79.40±2.35bc | 38.00 | 24.25±1.01bc |
| R |  |  |  |  |  |  |  |
| R1 | 209.14±11.19b | 30.31±0.63b | 94.72±10.73a | 48.66±2.74a | 275.67±1.55abc | 94.90 | 28.59±2.03ab |
| R2 | 195.09±6.47dc | 31.29±0.54b | 69.74±8.83cd | 46.81±0.13ab | 268.32±7.13bcd | 87.65 | 26.20±1.28bc |
| R3 | 207.06±7.73bc | 30.21±1.07b | 76.89±10.17bc | 46.21±2.78abc | 282.83±1.18ab | 90.39 | 28.31±2.18ab |
| R4 | 176.89±0.94e | 24.83±0.80d | 53.86±5.25e | 40.16±0.77e | 259.49±5.88cd | 75.70 | 21.05±1.67d |
| R5 | 201.24±7.31bc | 24.83±0.80d | 72.95±0.86bcd | 44.79±0.67bc | 262.55±9.24cd | 84.02 | 26.48±1.63abc |
| R6 | 199.15±5.48bc | 26.61±0.84d | 63.19±4.24cde | 43.35±0.72cd | 258.35±11.57d | 81.88 | 26.26±1.02bc |
| R7 | 223.76±5.56a | 35.66±1.22a | 85.52±7.95ab | 46.11±0.56abc | 287.09±8.68a | 97.01 | 30.64±0.48a |
| R8 | 182.57±0.82de | 24.08±0.02d | 72.84±7.19bcd | 39.72±1.68e | 256.83±16.61d | 79.42 | 23.79±0.55cd |
| R9 | 182.53±7.77de | 24.82±0.99d | 69.14±2.32cd | 41.38±0.72de | 262.08±2.28cd | 80.10 | 21.93±4.98d |
| R10 | 178.13±2.45e | 20.38±0.72e | 61.13±5.60de | 35.31±0.63f | 232.30±3.55e | 70.96 | 21.74±1.47d |

**Table. S2** Sensor performance description

| Array sequence number | Sensor name | Performance specification |
| --- | --- | --- |
| 1 | W1C | sensitive to aromatic components, benzene |
| 2 | W5S | highly sensitive to nitrogen oxides |
| 3 | W3C | sensitive to ammonia |
| 4 | W6S | sensitive to hydrides |
| 5 | W5C | sensitive to alkane aromatic components |
| 6 | W1S | sensitive to methane |
| 7 | W1W | sensitive to sulfides |
| 8 | W2S | sensitive to alcohols, aldehydes and ketones |
| 9 | W2W | sensitive to organic sulfides |
| 10 | W3S | sensitive to long chain alkanes |

**Table. S3** Details of 20 batches of samples

| Samples | | Source | Date of collection |
| --- | --- | --- | --- |
| H |  |  |  |
|  | H1 | Guangxi province | 2024.4.14 |
|  | H2 | Guangdong province | 2024.4.11 |
|  | H3 | Guangdong province | 2024.4.13 |
|  | H4 | Guangdong province | 2024.4.11 |
|  | H5 | Guangdong province | 2024.4.12 |
|  | H6 | Guangdong province | 2024.4.11 |
|  | H7 | Guangxi province | 2024.4.12 |
|  | H8 | Yunnan Province | 2024.4.12 |
|  | H9 | Guangxi province | 2024.4.11 |
|  | H10 | Yunnan Province | 2024.4.11 |
| R |  |  |  |
|  | R1 | Guangxi province | 2024.4.14 |
|  | R2 | Guangxi province | 2024.4.11 |
|  | R3 | Guangdong province | 2024.4.13 |
|  | R4 | Guangdong province | 2024.4.11 |
|  | R5 | Guangxi province | 2024.4.12 |
|  | R6 | Guangxi province | 2024.4.11 |
|  | R7 | Guangxi province | 2024.4.12 |
|  | R8 | Guangxi province | 2024.4.12 |
|  | R9 | Yunnan Province | 2024.4.11 |
|  | R10 | Guangxi province | 2024.4.12 |


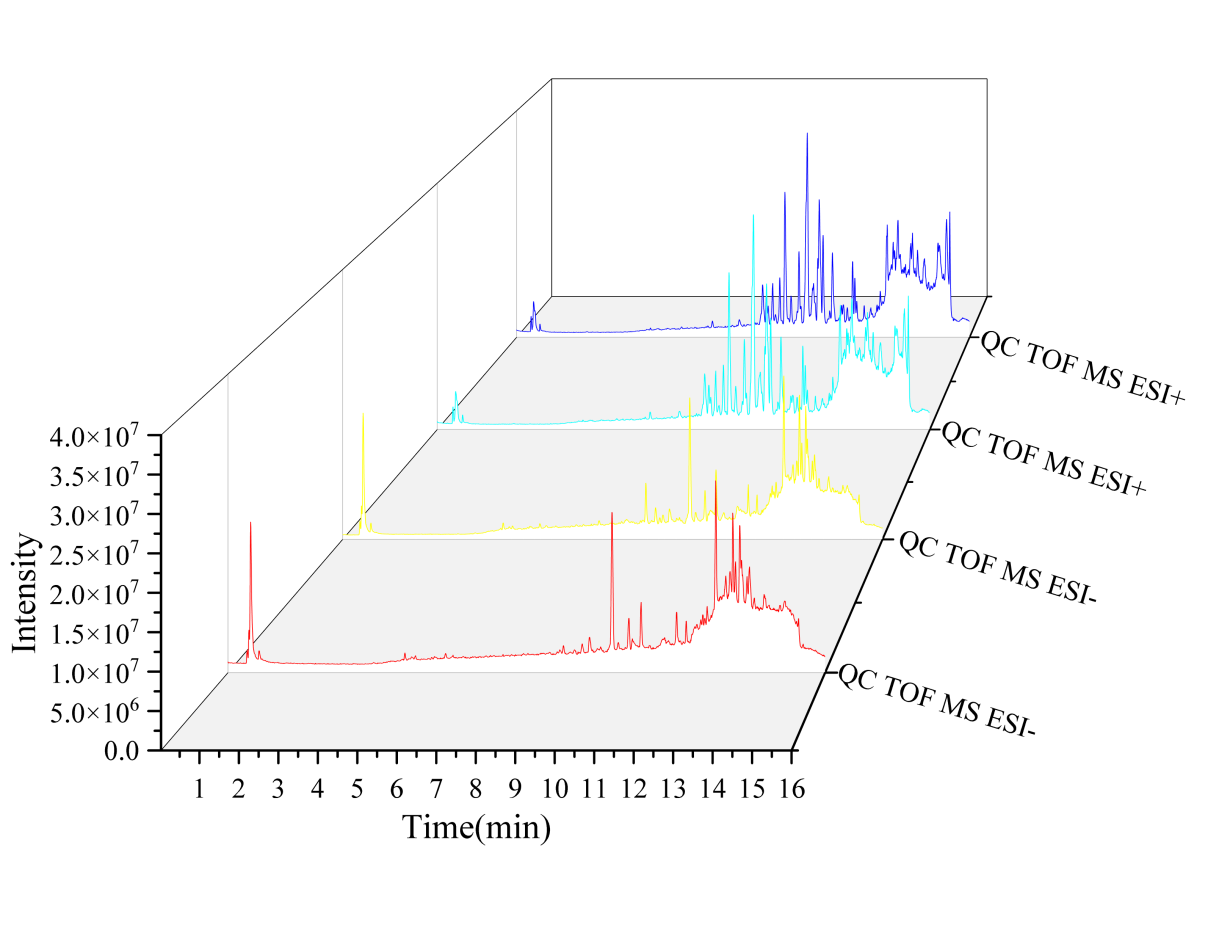


**Fig. S1** Total ion chromatogram (TIC) of the QC sample from positive and negative ESI modes.

**
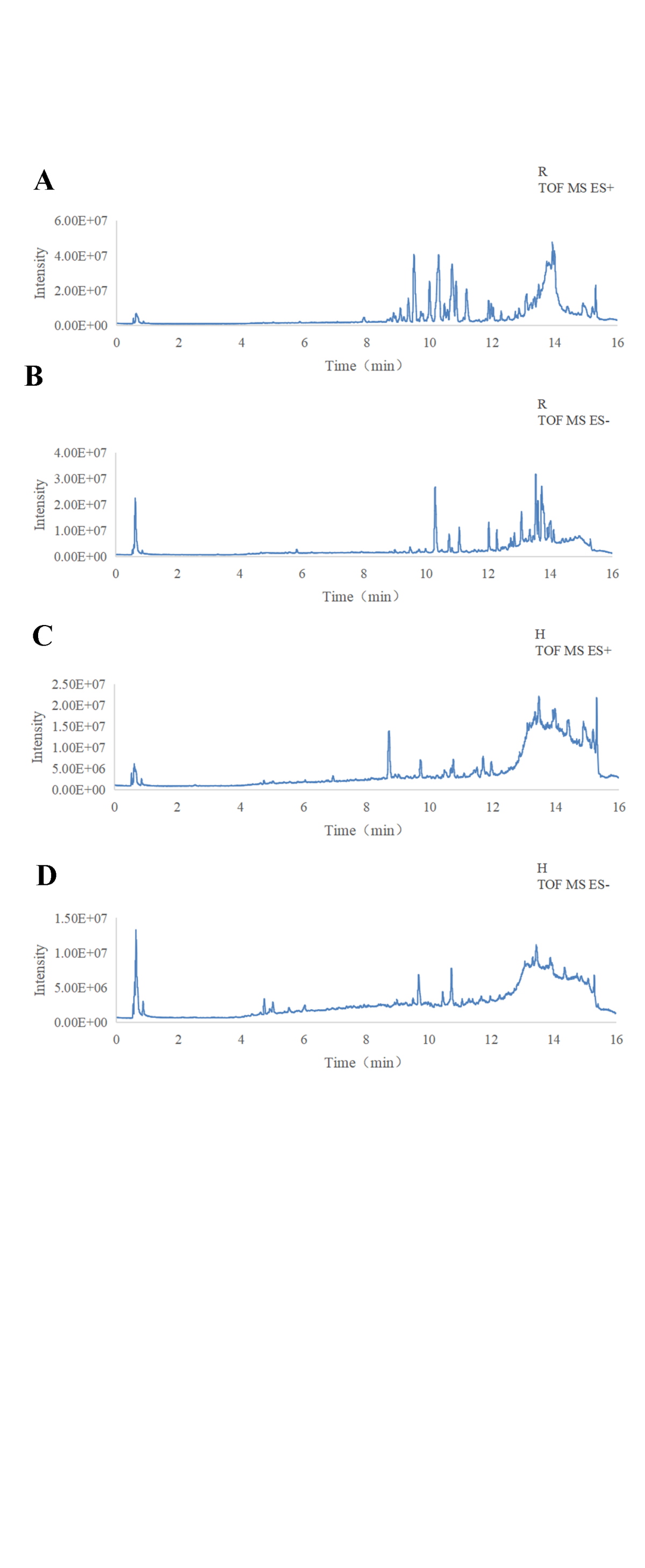
**

**Fig. S2** The representative total ion chromatogram (TIC) of R was acquired in positive (ESI+) (A) and negative (ESI−) (B) ionization modes; The representative total ion chromatogram (TIC) of H was acquired in positive (ESI+) (C) and negative (ESI−) (D) ionization modes.


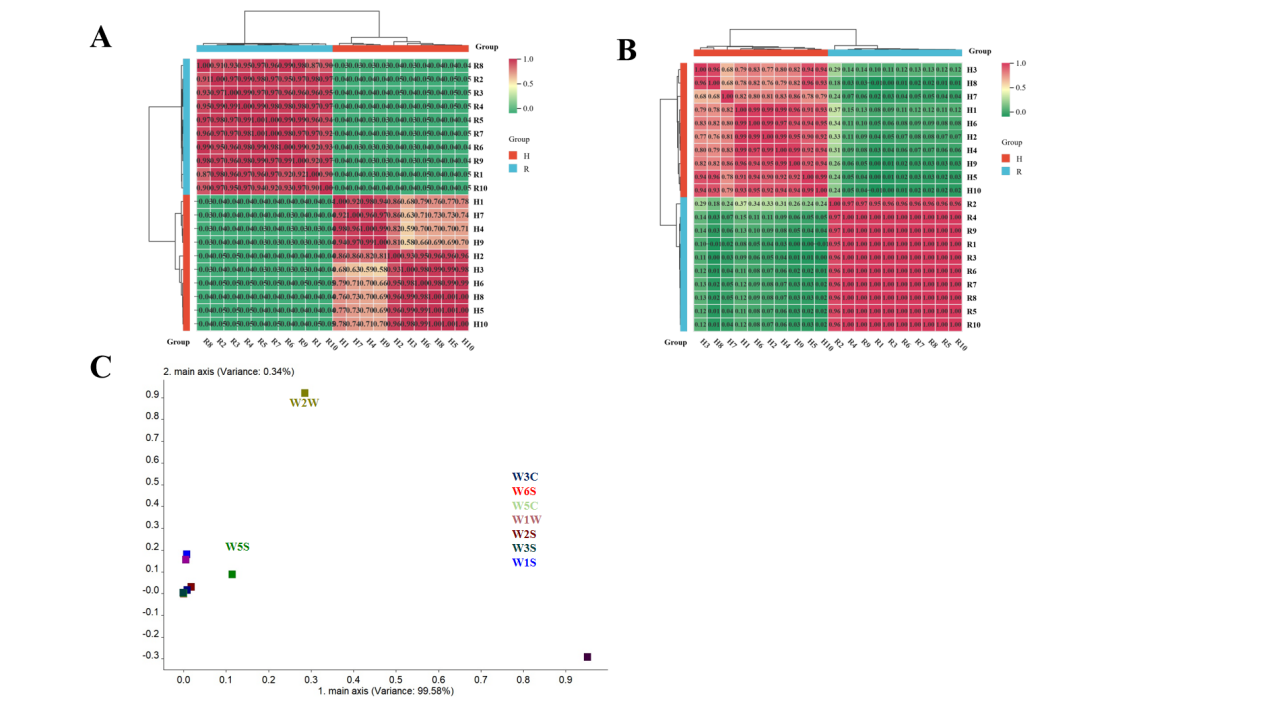


**Fig. S3** Intra-group correlation diagram of H and R in the positive ion mode in metabolomics (A); Intra-group correlation diagram of H and R in the positive ion mode in metabolomics (B);Loading diagram of electronic nose(C).


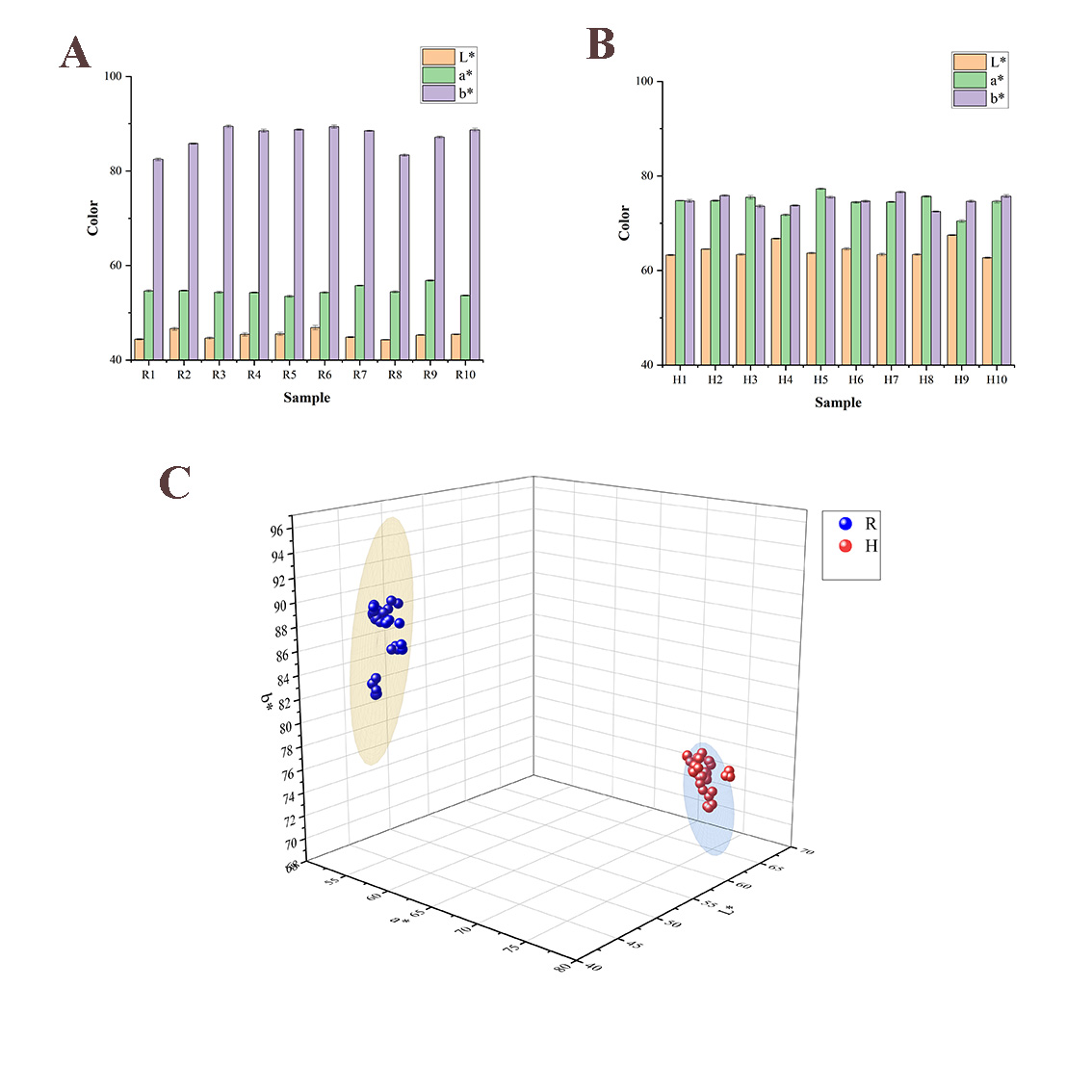


**Fig. S4** Error bar charts of L*, a*, and b* color values for Group R (A); error bar charts of L*, a*, and b* color values for Group H (B); 3D scatter plot of color parameters (L*, a*, b*) for Group R and Group H (C).


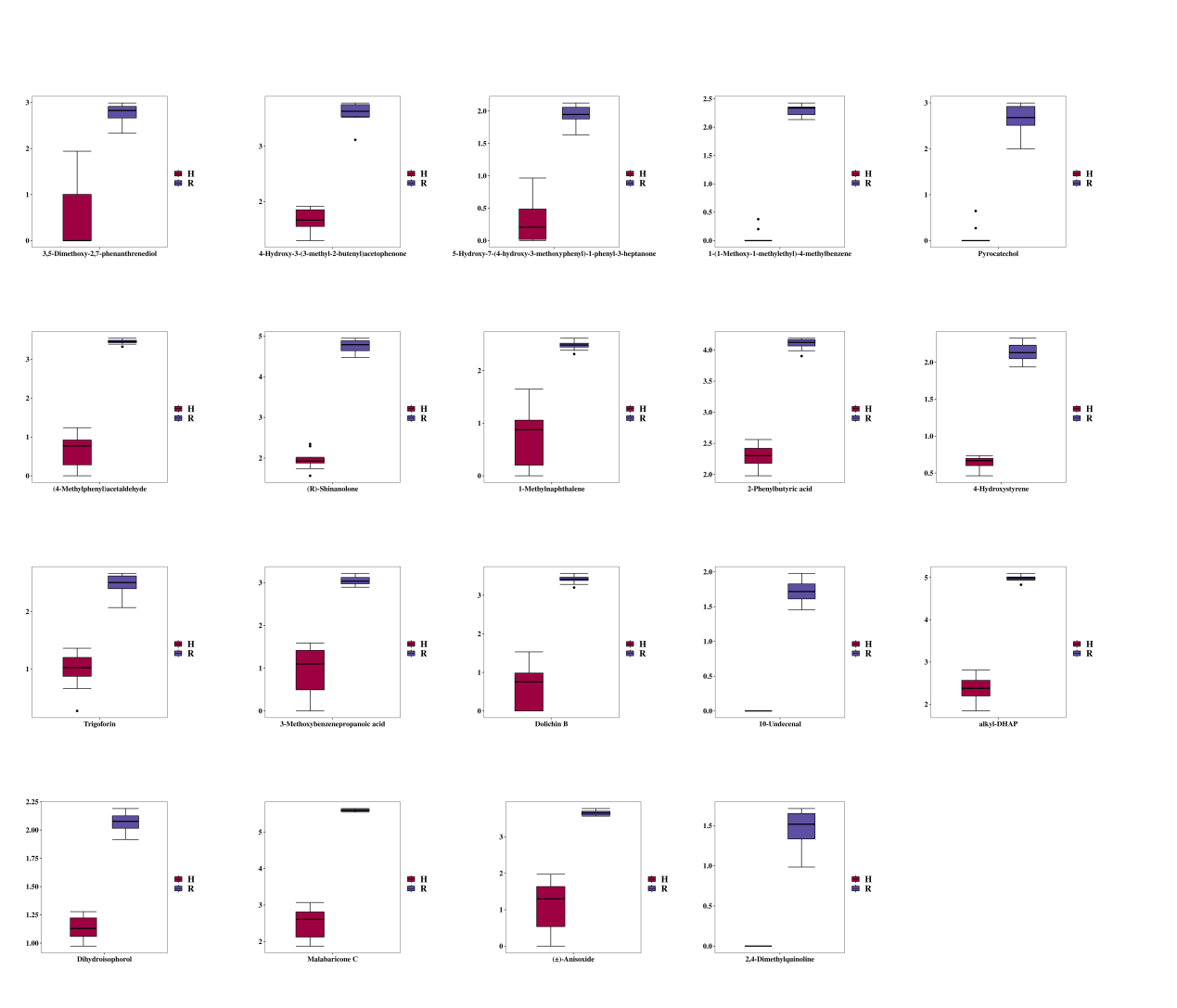


**Fig. S5** Box plots of the relative contents of 19 differential compounds in group H and group R.


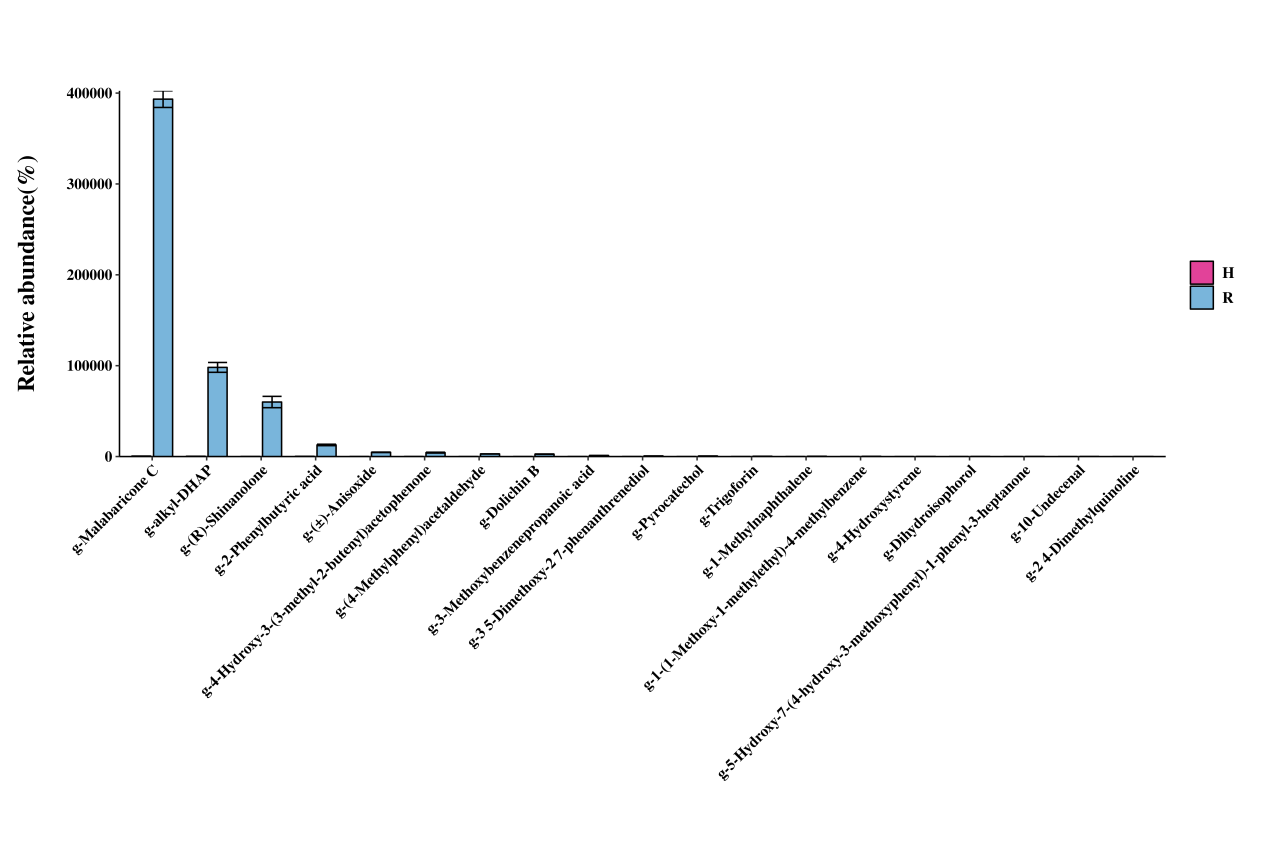


**Fig. S6** Bar charts of the relative abundances of 19 differential compounds in group H and group R. "g-" is a requirement for graphing and is not the name of a substance.
